# Draft Genome Sequences of Two *Fabibacter* sp. Strains Isolated from Coastal Surface Water of Aburatsubo Inlet, Japan

**DOI:** 10.1128/genomeA.01360-16

**Published:** 2016-12-08

**Authors:** Ryo Kaneko, Shu-Kuan Wong, Yoshitoshi Ogura, Tetsuya Hayashi, Susumu Yoshizawa, Koji Hamasaki

**Affiliations:** a Department of Marine Ecosystem Dynamics, Division of Marine Life Science, Atmosphere and Ocean Research Institute, University of Tokyo, Kashiwa, Chiba, Japan; b Department of Bacteriology, Faculty of Medical Sciences, Kyushu University, Fukuoka, Japan

## Abstract

Here, we report the draft genome sequences of *Fabibacter* sp. strain 4D4 and *F. misakiensis* strain SK-8^T^, isolated from surface seawater of a semienclosed inlet.

## GENOME ANNOUNCEMENT

The genus *Fabibacter*, a member of the family *Flammeovirgaceae* in the order *Cytophagales*, comprises three different species (1–3). To date, the type strains of all species from the genus were isolated mainly from marine environments such as marine sponge and seawater. In this study, the draft genomes of two strains, *Fabibacter* sp. strain 4D4 and *F. misakiensis* strain SK-8^T^ (3), isolated from seawater of Aburatsubo Inlet, Misaki, Japan, were sequenced.

Strain 4D4 was isolated from the sea surface microlayer (water depth of 36 µm) using the dram sampler method (4), while strain SK-8^T^ was isolated from the underlying water (water depth of 20 cm) near the pier of the Misaki Marine Biological Station, University of Tokyo (358°09.59′ N 139°36.59′ E). These two strains were isolated using methods described previously (3). Genomic DNA was extracted using the phenol-chloroform extraction and ethanol precipitation methods (5), and the draft genome sequencing was performed as previously reported (6). A total of 1,303,104 mate-paired and 1,109,892 paired-end reads from strain 4D4 and a total of 987,902 mate-paired and 1,812,416 paired-end reads from strain SK-8^T^ were assembled using Platanus version 1.2.4 (7). The sequences were annotated automatically using the RAST annotation system version 2.0 (http://rast.nmpdr.org). The calculation of the average nucleotide identity (ANI) was performed using the online ANI calculator (http://enve-omics.ce.gatech.edu/ani).

The 16S rRNA gene sequences were subjected to NCBI BLAST analysis, and *Fabibacter* sp. strain 4D4 shared the highest similarity to *F. misakiensis* strain SK-8^T^ (97.0% identity). The estimated genome sizes of strains 4D4 and SK-8^T^ were 4,577,373 bp (*N*_50_ length of 4,576,104 bp, G+C 41.7%) and 4,452,385 bp (*N*_50_ length of 1,336,118 bp, G+C 40.1%), respectively. These two strains shared a 76.41% ANI value. The draft genome assembly of strain 4D4 consisted of four scaffolds (average coverage of 114.8×), while the genome sequences of strain SK-8^T^ had five scaffolds (average coverage of 70.9×). The RAST annotation identified 4,092 coding sequences (CDSs) and 355 subsystems in the genome sequence of strain 4D4 and 3,888 CDSs and 350 subsystems in the genome sequence of strain SK-8^T^.

Strains 4D4 and SK-8^T^ possessed gene homologs involved in cell-wall and capsule subsystems, resistance to antibiotics and toxic compounds, inorganic sulfur assimilation, ammonia assimilation, CO_2_ fixation, dormancy and sporulation, and stress response. However, gene homologs from subsystems related to denitrification were observed only in the draft genome sequences of strain 4D4.

The draft genome information of these two strains may be able to provide insight into the potential roles of these microorganisms in surface seawater.

### Accession number(s).

The whole-genome shotgun projects of *Fabibacter* sp. strain 4D4 and *F. misakiensis* strain SK-8^T^ have been deposited in DDBJ/EMBL/GenBank under the accession numbers MDGP00000000 and MDGQ00000000, respectively. The versions described in this paper are the first versions, MDGP00100000 and MDGQ01000000.
